# A Prospective Observational Study of Anamorelin During Chemoimmunotherapy in Non‐Small Cell Lung Cancer With Cachexia (SPIRAL‐ANA)

**DOI:** 10.1002/jcsm.70340

**Published:** 2026-07-07

**Authors:** Kenji Morimoto, Junji Uchino, Makoto Hibino, Yuki Takeyasu, Ken Yamamoto, Yoshie Morimoto, Yusuke Chihara, Takayuki Nakano, Hiroyasu Kaneda, Shunya Tanaka, Shinsuke Ogusu, Yasuhiro Goto, Takashi Nomizo, Takayo Ota, Hiroaki Tsukamoto, Yoshizumi Takemura, Katsumi Nakatomi, Hayato Koba, Kohei Yoshimine, Takayuki Shimose, Tadaaki Yamada, Koichi Takayama

**Affiliations:** ^1^ Department of Pulmonary Medicine Kyoto Prefectural University of Medicine Kyoto Japan; ^2^ Department of Respiratory Medicine Shonan Oiso Hospital Kanagawa Japan; ^3^ Department of Respiratory Medicine Shonan Fujisawa Tokushukai Hospital Kanagawa Japan; ^4^ Department of Thoracic Oncology Kansai Medical University Osaka Japan; ^5^ Division of Thoracic Oncology Kobe Minimally Invasive Cancer Center Hyogo Japan; ^6^ Department of Pulmonary Medicine Kyoto Kuramaguchi Medical Center Kyoto Japan; ^7^ Department of Respiratory Medicine Uji‐Tokushukai Medical Center Kyoto Japan; ^8^ Department of Respiratory Medicine Fukuchiyama City Hospital Kyoto Japan; ^9^ Department of Clinical Oncology, Graduate School of Medicine Osaka Metropolitan University Osaka Japan; ^10^ Department of Respiratory Medicine Japanese Red Cross Society Kyoto Daiichi Hospital Kyoto Japan; ^11^ Division of Hematology, Respiratory Medicine and Oncology, Department of Internal Medicine, Faculty of Medicine Saga University Saga Japan; ^12^ Department of Respiratory Medicine Fujita Health University School of Medicine Aichi Japan; ^13^ Department of Respiratory Medicine Kyoto University Graduate School of Medicine Kyoto Japan; ^14^ Department of Breast Medical Oncology Izumi City General Hospital Osaka Japan; ^15^ Department of Respiratory Medicine National Hospital Organization Himeji Medical Center Hyogo Japan; ^16^ Department of Pulmonary Medicine Otsu City Hospital Shiga Japan; ^17^ Department of Respiratory Medicine National Hospital Organization Ureshino Medical Center Saga Japan; ^18^ Department of Respiratory Medicine Kanazawa University Hospital Kanazawa Japan; ^19^ Department of Respiratory Medicine Iizuka Hospital Fukuoka Japan; ^20^ Statistics and Data Center, Clinical Research Support Center Kyushu Fukuoka Japan

**Keywords:** anamorelin, cancer cachexia, chemoimmunotherapy, non‐small cell lung cancer

## Abstract

**Background:**

The prognosis of patients with advanced non‐small cell lung cancer (NSCLC) complicated by cancer cachexia remains poor. Anamorelin, a ghrelin receptor agonist, has been approved only in Japan for the treatment of cancer cachexia.

**Methods:**

This prospective multicentre observational study was conducted to evaluate the impact of anamorelin administration on the efficacy and safety of chemoimmunotherapy in patients with NSCLC and cancer cachexia. The primary endpoint was progression‐free survival (PFS), evaluated against a predefined threshold derived from a previous retrospective cohort of patients with cachectic NSCLC treated with chemoimmunotherapy alone. This study was registered in the Japan Registry of Clinical Trials (jRCT1071210053).

**Results:**

Between August 2021 and January 2024, 123 patients were enrolled from 29 institutions in Japan, with 114 included in the full analysis set. The median age was 73 years; 28 (24.6%) were female and 20 patients (17.5%) had a performance status of 2. The objective response rate was 57.9% (95% confidence interval [CI]: 48.7%–66.6%). Median PFS and overall survival (OS) were 6.2 months (95% CI: 4.6–7.7) and 18.5 months (95% CI: 13.1–28.6), respectively. The lower limit of the 95% CI for PFS did not exceed the predefined threshold of 5 months. At week 12, 61 of 85 patients (71.8%) had transitioned from cancer cachexia to a non‐cachectic state. Landmark survival analyses at week 12 showed that patients who achieved this transition had significantly longer OS compared with those who remained cachectic (19.9 vs. 7.1 months; hazard ratio for cachexia vs. transition, 2.19; 95% CI: 1.19–4.02; *p* = 0.0098). Grade ≥ 3 hyperglycemia was observed in six patients (5.1%), but no new safety signals were identified.

**Conclusions:**

Anamorelin combined with chemoimmunotherapy did not prolong PFS in advanced NSCLC with cancer cachexia, but achieved a high rate of cachexia reversal. Transition to a non‐cachectic state was associated with improved OS, suggesting that cachexia reversal may serve as a surrogate for survival benefit.

**Trial Registration:**

jRCT1071210053 (jRCT).

## 1 Introduction

Lung cancer is one of the leading causes of cancer‐related mortality worldwide, and non‐small cell lung cancer (NSCLC) accounts for the majority of cases [1]. Despite recent advances in systemic therapy, the prognosis for patients with advanced NSCLC has historically been poor. In recent years, multiple phase III trials including treatment‐naïve patients with NSCLC have demonstrated that chemoimmunotherapy, the combination of platinum‐based chemotherapy and immune checkpoint inhibitors (ICIs), improves progression‐free survival (PFS) and overall survival (OS) compared with chemotherapy alone [2, 3, 4, 5]. Based on these findings, chemoimmunotherapy is now recommended as the standard first‐line treatment for patients with advanced‐stage or postoperative recurrent NSCLC without oncogenic driver mutations. After its approval as a first‐line treatment for advanced NSCLC, chemoimmunotherapy is now administered to patients with diverse clinical backgrounds, including those with cancer cachexia [6]. Despite the established efficacy of chemoimmunotherapy, clinical outcomes in patients with cancer cachexia remain unsatisfactory. Retrospective studies reported that cachectic patients showed significantly shorter PFS and OS compared with non‐cachectic ones [7, 8, 9]. Anamorelin, the first and only drug approved in Japan for the treatment of cancer cachexia, is a ghrelin receptor agonist that has consistently been shown in clinical studies to increase lean body mass and improve appetite [10, 11]. The ROMANA study, a phase III clinical trial on anamorelin conducted in Europe, confirmed these results [12]. By ameliorating cancer cachexia, anamorelin may enhance the efficacy of chemoimmunotherapy; however, whether this translates into improved clinical outcomes in this subpopulation remains unclear.

To our knowledge, this is the first prospective, non‐randomized study to evaluate the efficacy and safety of anamorelin in patients with NSCLC undergoing chemoimmunotherapy. This study aims to clarify the potential clinical benefits of combining anamorelin with standard first‐line treatment in this challenging patient subpopulation.

## 2 Methods

### 2.1 Study Design and Treatment

The SPIRAL‐ANA was a prospective observational study. We evaluated the impact of anamorelin administration on the efficacy and safety of chemoimmunotherapy in patients with NSCLC and cancer cachexia. The primary endpoint was PFS. The secondary endpoints were treatment‐emergent adverse events (AEs); best overall response (BOR); time to treatment failure (TTF); OS; BOR, TTF, PFS and OS according to programmed death‐ligand 1 (PD‐L1) expression status; changes in body weight, ECOG performance status (ECOG‐PS) and Quality of Life (QOL) Questionnaire for Cancer Patients Treated with Anticancer Drugs (QOL‐ACD) scores at 3, 12 and 24 weeks after initiation of anamorelin; the continuation rate of anamorelin at 12 weeks; and the prevalence of cancer cachexia at 12 and 24 weeks. The QOL‐ACD is a self‐rated scale developed to evaluate the QOL of Japanese patients receiving chemotherapy, consisting of four domains (functional, physical, psychological and social) as well as a global face scale [10, 11]. Missing data at each time point were excluded from the corresponding analyses. Anamorelin was administered orally at a dose of 100 mg once daily. Chemoimmunotherapy included platinum‐based regimens combined with ICIs (e.g., pembrolizumab or atezolizumab), administered every 3 weeks according to standard clinical practice; nivolumab plus ipilimumab was allowed in a minority of cases. The distribution of treatment regimens is shown in Table S1. Nutritional counselling by dietitians was provided according to routine clinical practice at the discretion of each institution.

### 2.2 Ethical Considerations

This study was approved by the Nonprofit Organization Clinical Trial Network Fukuoka on 4 August 2021 (approval number: 21‐E02). In accordance with the Declaration of Helsinki, written informed consent was obtained from all patients prior to enrolment.

### 2.3 Study Participants, Eligibility and Exclusion Criteria

Patients were enrolled between August 2021 and January 2024 and followed up until 31 January 2025. The complete inclusion and exclusion criteria are provided in Table S2. Briefly, eligible patients had unresectable stage III or IV or postoperative recurrent NSCLC associated with cancer cachexia for which anamorelin was indicated and had been scheduled to receive chemoimmunotherapy. In Japan, anamorelin is indicated for patients with body weight loss of ≥ 5% within the previous 6 months and with anorexia, who meet at least two of the following criteria: (1) fatigue or malaise, (2) generalized muscle weakness or (3) C‐reactive protein (CRP) level > 0.5 mg/dL, albumin level < 3.2 g/dL or haemoglobin level < 12 g/dL. The clinical indication for anamorelin is broadly comparable to Evans' criteria [13].

### 2.4 Cancer Cachexia

Baseline cancer cachexia was defined according to the indication criteria for anamorelin in Japan. Cancer cachexia at weeks 12 and 24 was assessed using the same criteria as at baseline, incorporating clinical symptoms and laboratory parameters. Body weight loss exceeding 5% was calculated as follows: (maximum body weight within 6 months prior to treatment initiation—body weight at week X) / maximum body weight within 6 months prior to treatment initiation × 100, where X indicates week 12 or week 24.

### 2.5 Tumour Assessment

Tumour assessment of the chest and abdomen was conducted via computed tomography (CT) at baseline and approximately every 8 to 12 weeks thereafter. Intracranial magnetic resonance imaging was performed with the same frequency, particularly when disease progression was suspected. Tumour response was evaluated using RECIST, version 1.1. CT images at enrolment were used as the baseline. BOR was defined as the best overall treatment response observed during the treatment period. Objective response rate (ORR) was defined as the proportion of patients who achieved a complete or partial response as the BOR. PFS was defined as the total time elapsed from enrolment to confirmation of disease progression or death for any cause. OS was defined as the total time elapsed from enrolment to death for any cause.

### 2.6 Sample Size Calculation

In a previous retrospective study, the median PFS for patients with NSCLC and cancer cachexia, defined as body weight loss > 5% with laboratory abnormalities, was 6.7 months (95% confidence interval [CI]: 4.6–9.5 months), and this subgroup was used as the basis for defining the predefined threshold for the primary endpoint [7]. Considering the additional impact of symptoms such as anorexia and muscle weakness, the actual PFS is expected to be lower. Therefore, we set the threshold PFS at 5.0 months. In the aforementioned retrospective study, the median PFS in the non‐cachexia group was 8.9 months; however, pivotal prospective clinical trials have reported a range of 6.7–9.0 months depending on the regimen, with cachexia status and proportion of patients with cachexia not reported [2, 3, 4, 5]. Taking into account the additional burden of anorexia and muscle weakness, we set the expected median PFS for patients treated with anamorelin at 7.5 months. Assuming an enrolment period of 2 years, a follow‐up period of 12 months, a two‐sided α of 0.05 and a power of 0.8, the required sample size was estimated to be 114 patients. Considering a 10% dropout rate, the target sample size was estimated to be 127 patients.

### 2.7 Analysis of PD‐L1 Expression

PD‐L1 expression was evaluated using the PD‐L1 IHC 22C3 pharmDx assay (Dako/Agilent, Santa Clara, CA, USA). Tumoral PD‐L1 expression was calculated as the percentage of cells showing complete or partial membrane staining among at least 100 viable tumour cells. The cutoff value for PD‐L1 status was set at 50% based on previous studies [7, 14, 15].

### 2.8 Statistical Analysis

PFS and OS were estimated using the Kaplan–Meier method, and median values with their 95% CIs were calculated. CIs were estimated using the method of Brookmeyer and Crowley. The protocol treatment was considered effective if the lower limit of the 95% CI for median PFS exceeded 5 months. The BOR and corresponding two‐sided 95% CIs were estimated using the Wilson method. Group comparisons for binary variables were performed using the chi‐square test, and comparisons for time‐to‐event variables were performed using the log‐rank test and Cox proportional hazards model. Logistic regression analyses were performed to identify baseline factors associated with the presence of cancer cachexia at weeks 12 and 24. All statistical tests were two‐sided, with a significance level of 5%, and 95% CIs were calculated without adjustment for multiplicity.

For the safety analysis, AEs were summarized according to Common Terminology Criteria for Adverse Events version 5.0 in the safety analysis set (SAS). Continuous variables are presented as medians with ranges, and categorical variables as counts and percentages.

The full analysis set (FAS) was defined as all patients enrolled in the study who received at least one dose of chemoimmunotherapy and anamorelin. All efficacy endpoints were determined using the FAS. Statistical analyses were performed using SAS version 9.4 (SAS Institute Inc., Cary, NC, USA). The statistical analysis plan was developed and reviewed by the responsible biostatistician, Takayuki Shimose.

## 3 Results

### 3.1 Patient Demographics

**FIGURE 1:**
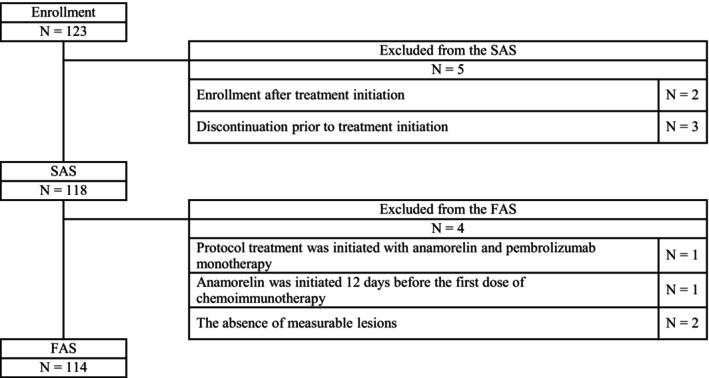
Patient flow diagram. FAS: full analysis set; SAS: safety analysis set.

Between September 2021 and January 2024, a total of 123 patients were enrolled from 29 institutions in Japan, of whom 118 were included in the SAS and 114 in the FAS (Figure 1). In the FAS, median age, body weight and body mass index were 73 years (range: 42–89 years), 53.2 kg (range: 32.5–79.4 kg) and 20.2 kg/m^2^ (range: 15.0–29.1 kg/m^2^), respectively. Eighty‐six patients (75.4%) were male. In total, 52 patients (45.6%) had adenocarcinoma, 96 (84.2%) had stage IV disease, 6 (5.3%) had an epidermal growth factor receptor (EGFR) mutation and 20 (17.5%) had an ECOG‐PS score of 2. High PD‐L1 expression (≥ 50%) was observed in 37 patients (32.5%), whereas low PD‐L1 expression (< 50%) was observed in 73 patients (64.0%) (Table 1). Details of the chemoimmunotherapy regimens are provided in Table S1.

**TABLE 1:** Baseline characteristics of the study population.

Item	Category	FAS
Sex	Male	86 (75.4%)
	Female	28 (24.6%)
Age	*N*	114
	Median (min–max)	73.0 (42.0–89.0)
BW (kg)	*N*	114
	Median (min–max)	53.2 (32.5–79.4)
BMI (kg/m^2^)	*N*	114
	Median (min–max)	20.2 (15.0–29.1)
Histology	Adenocarcinoma	52 (45.6%)
	Squamous cell carcinoma	41 (36.0%)
	Others	21 (18.4%)
Stage	III	8 (7.0%)
	IV	96 (84.2%)
	Postoperative recurrence	10 (8.8%)
Driver mutation	*EGFR* mutation	6 (5.3%)
Smoking status	Never smoker	18 (15.8%)
	Current smoker	30 (26.3%)
	Former smoker	66 (57.9%)
PD‐L1 TPS	< 50%	73 (64.0%)
	≥ 50%	37 (32.5%)
	Not tested	4 (3.5%)
ECOG‐PS	0	13 (11.4%)
	1	81 (71.1%)
	2	20 (17.5%)
Baseline total tumour size (mm)	*N*	114
	Median (min–max)	61.2 (10.0–260.0)

Abbreviations: BMI, body mass index; BW, body weight; ECOG‐PS, Eastern Cooperative Oncology Group performance status; EGFR, epithelial growth factor receptor; FAS, full analysis set; PD‐L1, programmed death‐ligand 1; TPS, tumour proportion score.

### 3.2 QOL‐ACD Score

For Item 8 (‘Did you have a good appetite?’), patient‐reported outcomes showed significant improvements from baseline at weeks 3, 12 and 24, consistent with the findings of previous clinical trials (*p* = 0.002, *p* = 0.031 and *p* = 0.012, respectively) [10, 11, 12]. Furthermore, the outcomes for Item 9 suggest not only an improvement in appetite, but also that patients were able to enjoy meals at weeks 3 and 12 (*p* = 0.086 and *p* = 0.084, respectively). In addition, for Item 22 (‘face scale’), scores showed significant improvements from baseline at weeks 3 and 12 (*p* = 0.006 and *p* = 0.029, respectively). However, scores in the social attitude domain significantly declined compared with baseline at weeks 3, 12 and 24 (*p* < 0.001, *p* = 0.007 and *p* = 0.004, respectively) (Figure S1).

### 3.3 Body Weight, Cachexia‐Related Laboratory Values and ECOG‐PS

There were no significant changes in body weight at 3, 12 or 24 weeks compared with baseline (*p* = 0.923, *p* = 0.501 and *p* = 0.965, respectively) (Figure S2).

Figure S2 also shows the evolution of cachexia‐related laboratory values: haemoglobin, albumin and C‐reactive protein. Haemoglobin levels decreased over time at 3 and 12 weeks (*p* < 0.001, *p* < 0.001, respectively), and there was no significant change from baseline at 24 weeks (*p* = 0.238). Albumin levels were significantly higher than at baseline at 12 and 24 weeks (*p* = 0.002 and *p* < 0.001, respectively). C‐reactive protein levels were significantly lower than those at baseline at 3, 12 and 24 weeks (*p* < 0.001 for all comparisons). By week 24, although deterioration in ECOG‐PS was observed in a small subset of patients, the majority showed scores within the 0–2 range (Table S3).

### 3.4 Duration of Anamorelin Treatment and Reasons for Discontinuation

The median duration of anamorelin treatment was 13.15 weeks. At week 12, 62 patients (54.4%) were still being treated with anamorelin. The reasons for discontinuation are summarized in Figure S3. The most common reason for discontinuation was disease progression or discontinuation of chemoimmunotherapy (47.4%). Within the first 12 weeks, the leading cause was AEs, including those chemoimmunotherapy‐related (38.0%), whereas after 12 weeks it was disease progression or discontinuation of chemoimmunotherapy (58.0%).

### 3.5 Efficacy

The ORR of chemoimmunotherapy was 57.9% (48.7%–66.6%). The overall responses of chemoimmunotherapy were as follows: complete response in one patient (0.9%), partial response in 65 (57.0%), stable disease in 26 (22.8%) and progressive disease in 18 (15.8%), whereas in four patients (3.5%) the response was not evaluable (Table S4).

**FIGURE 2:**
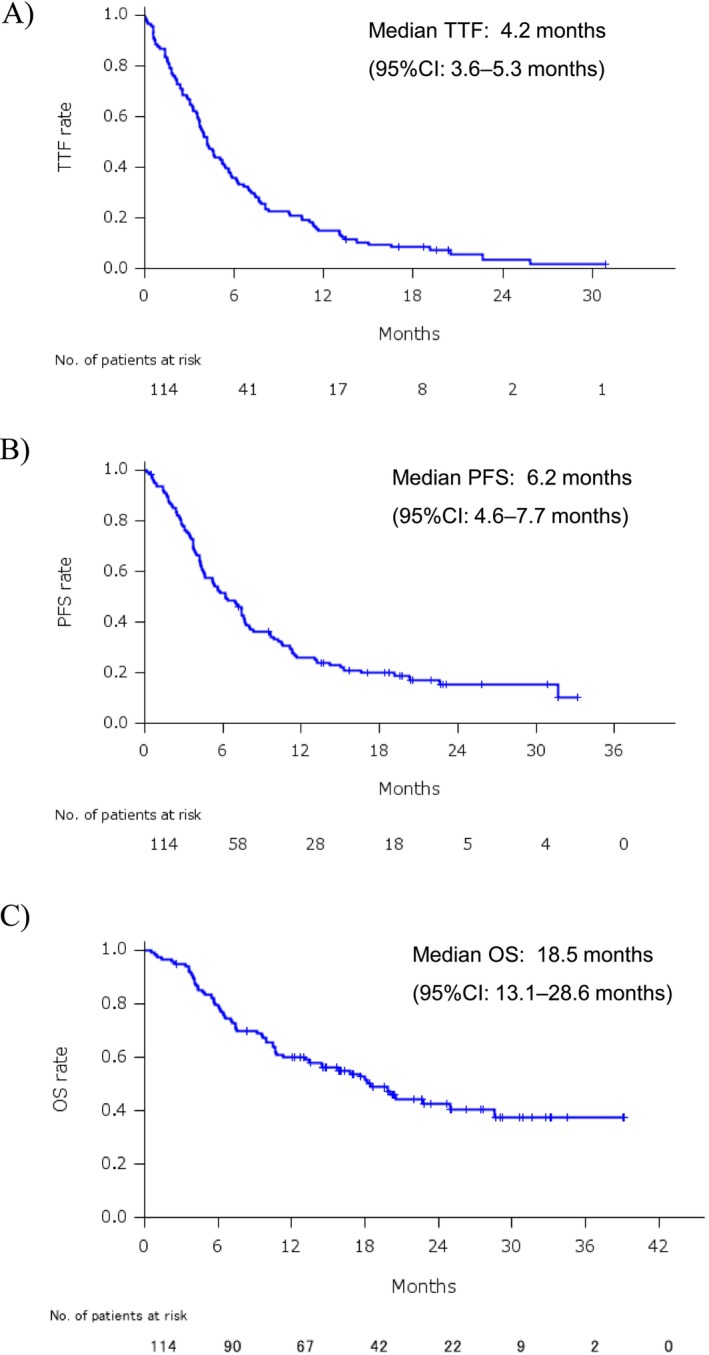
TTF, PFS, and OS of the patients treated with anamorelin in combination with chemoimmunotherapy. Kaplan–Meier curves depicting (A) TTF, (B) PFS and (C) OS of patients who received anamorelin in combination with chemoimmunotherapy. CI, confidence interval; NE, not estimable; OS, overall survival; PFS, progression‐free survival; TTF, time to treatment failure.

The follow‐up period ranged from 0.6 to 39.1 months, with a median of 14.5 months. The median TTF was 4.2 months (95% CI: 3.6–5.3 months) (Figure 2A). The primary endpoint, median PFS, was 6.2 months (95% CI: 4.6–7.7 months), with the lower limit of the CI falling below the threshold value of 5.0 months (Figure 2B). The median OS was 18.5 months (95% CI: 13.1–28.6 months) (Figure 2C). At the study termination date, six patients were continuing chemoimmunotherapy and one had been transferred to another hospital with subsequent outcomes unknown. Of the remaining 107 patients, 58 (54.2%) received subsequent therapy.

Subgroup analyses were conducted according to PD‐L1 expression status. Patients with high PD‐L1 expression (≥ 50%) demonstrated a significantly higher ORR compared with those with low expression (< 50%) (75.7% vs. 50.7%, *p* = 0.012) (Table S5). As shown in Figure S4A–C, no significant differences in TTF, PFS or OS were observed between patients with high PD‐L1 expression and those with low expression (TTF: 5.4 vs. 4.0 months, *p* = 0.5342; PFS: 7.0 vs. 6.2 months, *p* = 0.3774; OS: 18.2 vs. 20.5 months, *p* = 0.8789). We performed a landmark analysis using 12 weeks as the cutoff for the duration of anamorelin treatment. There were no significant differences in PFS or OS between patients who received anamorelin for ≥ 12 weeks and those who received it for < 12 weeks (PFS: 4.8 months vs. 4.8 months, *p* = 0.6534; OS: 17.7 months vs. 15.4 months, *p* = 0.4462) (Figure S5A,B).

### 3.6 Impact of Cachexia Status Transition on Prognosis

**FIGURE 3:**
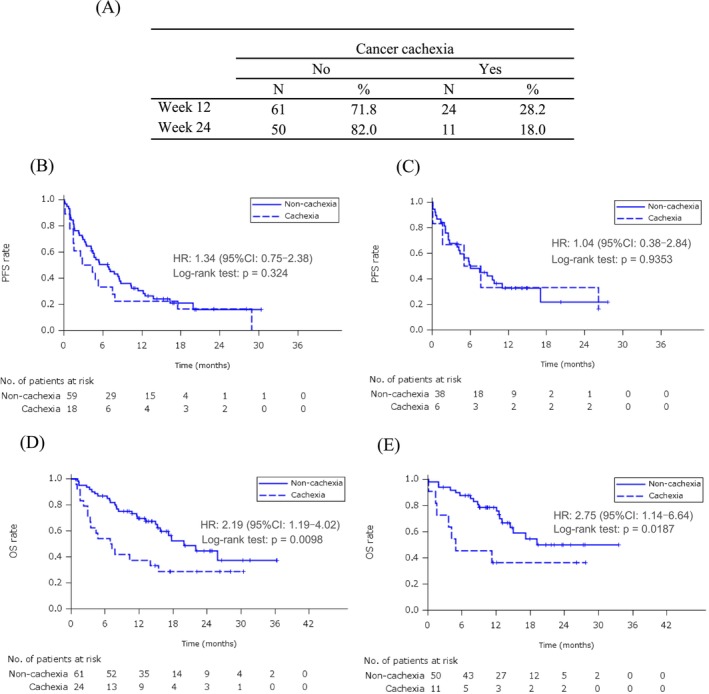
Distribution of cachexia status and landmark analyses of survival outcomes. (A) Distribution of cancer cachexia status at weeks 12 and 24. (B) Kaplan–Meier curves for PFS stratified by cachexia status at week 12. (C) Kaplan–Meier curves for PFS stratified by cachexia status at week 24. (D) Kaplan–Meier curves for OS stratified by cachexia status at week 12. (E) Kaplan–Meier curves for OS stratified by cachexia status at week 24. CI, confidence interval; HR, hazard ratio; OS, overall survival; PFS, progression‐free survival.

At week 12, 61 of 85 patients (71.8%) had transitioned from cancer cachexia to a non‐cachectic state, and at week 24, 50 of 61 patients (82.0%) had transitioned (Figure 3A). To address immortal time bias, we performed landmark survival analyses at each time point. In the landmark analysis of PFS at 12 and 24 weeks, no significant differences were observed between patients who had transitioned to a non‐cachectic state and those who remained cachectic (12‐week landmark: 6.7 months vs. 3.6 months, *p* = 0.324; 24‐week landmark: 6.0 months vs. 6.4 months, *p* = 0.9353) (Figure 3B,C). In contrast, patients who had transitioned to a non‐cachectic state showed a significantly longer OS compared with those who remained cachectic (12‐week landmark: 19.9 months vs. 7.1 months, *p* = 0.0098; 24‐week landmark: not reached vs. 5.0 months, *p* = 0.0187) (Figure 3D,E). We performed logistic regression analyses of baseline factors predictive of cancer cachexia at weeks 12 and 24. However, no predictive factors for cancer cachexia were identified at either time point (Tables S6 and S7).

### 3.7 Safety

The reasons for discontinuation of chemoimmunotherapy were as follows: disease progression or death in 69 patients (58.5%), AEs in 28 (23.7%), patient request to discontinue in seven (5.9%) and performance status deterioration in six (5.1%) (Figure S6). AEs for any cause in the SAS are shown in Table 2. The most common AE with Grade 3 or higher was neutropenia, which occurred in 37 patients (31.4%). Notably, hyperglycemia of Grade 3 or higher occurred in six patients (5.1%). Three patients died as a result of AEs: one from suicide and one from pneumonitis, whereas the cause of death for the third one was not specified. No deaths suggestive of being related to the anamorelin treatment were observed.

**TABLE 2:** Adverse events of any cause in the SAS^a^.

Event	Any grade (%)	Grade ≥ 3 (%)
Hypoalbuminemia	115 (97.5)	5 (4.2)
Anaemia	111 (94.1)	15 (12.7)
Malaise	103 (87.3)	11 (9.3)
Fatigue	99 (83.9)	10 (8.5)
Anorexia	93 (78.8)	12 (10.2)
Hyponatremia	87 (73.7)	5 (4.2)
Neutropenia	72 (61.0)	37 (31.4)
Muscle weakness: General	70 (59.3)	4 (3.4)
Gamma‐glutamyl transferase increased	64 (54.2)	8 (6.8)
Leukopenia	64 (54.2)	24 (20.3)
Aspartate aminotransferase increased	61 (51.7)	9 (7.6)
Dyspnea	59 (50.0)	9 (7.6)
Thrombocytopenia	58 (49.2)	4 (3.4)
Alanine aminotransferase increased	57 (48.3)	7 (5.9)
Blood creatinine increased	45 (38.1)	0 (0.0)
Hyperkalemia	42 (35.6)	0 (0.0)
Nausea	41 (34.7)	0 (0.0)
Alopecia	39 (33.1)	—
Proteinuria	37 (31.4)	0 (0.0)
Hypokalemia	36 (30.5)	5 (4.2)
Cough	33 (28.0)	0 (0.0)
Fever	32 (27.1)	0 (0.0)
Weight loss	32 (27.1)	1 (0.8)
Hypocalcemia	31 (26.3)	3 (2.5)
Hypercalcemia	27 (22.9)	1 (0.8)
Diarrhoea	26 (22.0)	0 (0.0)
Pneumonitis	19 (16.1)	3 (2.5)
Mucositis oral	18 (15.3)	0 (0.0)
Blood bilirubin increased	16 (13.6)	2 (1.7)
Constipation	16 (13.6)	0 (0.0)
Rash acneiform	16 (13.6)	1 (0.8)
Vomiting	15 (12.7)	0 (0.0)
Arthralgia	12 (10.2)	0 (0.0)
Peripheral sensory neuropathy	11 (9.3)	0 (0.0)
Edema limbs	9 (7.6)	0 (0.0)
Hyperglycemia	9 (7.6)	6 (5.1)
Hypernatremia	9 (7.6)	2 (1.7)
Lung infection	8 (6.8)	7 (5.9)
Myalgia	8 (6.8)	0 (0.0)
Dysgeusia	7 (5.9)	—

^a^ This table summarizes adverse events with an incidence of > 5% in the SAS. SAS, safety analysis set.

## 4 Discussion

Cancer cachexia is a common and debilitating condition in patients with advanced NSCLC. Cachexia is strongly associated with impaired QOL, reduced treatment tolerance and poor survival outcomes [6, 8]. Despite its clinical importance, there is no clear consensus on interventions aimed at reversing cachexia and improving survival [16]. Against this background, our prospective multicentre study provides new evidence on the potential use of anamorelin for this purpose when combined with chemoimmunotherapy.

In this study, the combination of anamorelin and chemoimmunotherapy did not significantly improve PFS. Although PFS is a conventional primary endpoint in first‐line NSCLC trials, it may not adequately reflect the benefits of anamorelin, which acts as a supportive agent to ameliorate cachexia rather than as an antitumour therapy. These findings suggest that the clinical value of anamorelin may lie more in improving cachexia‐related symptoms and patients' functional status rather than in direct tumour control. Importantly, in this context, cachexia reversal and improvements in patient‐reported outcomes represent clinically meaningful benefits beyond tumour response alone. Cancer cachexia itself has also been reported to attenuate the efficacy of ICIs, in agreement with our observation that treatment efficacy did not differ according to PD‐L1 expression levels [17]. In the present study, PD‐L1 expression ≥ 50% was observed in 32.5% of patients, indicating that a PD‐L1‐high population was not predominant [18]. In our previous retrospective study, a higher proportion of PD‐L1 expression ≥ 50% was observed in the cachectic subgroup; however, this pattern was not reproduced in the present prospective cohort [7]. Therefore, the association between PD‐L1 expression and cancer cachexia remains unclear and requires further validation.

The median OS in our cohort was 18.5 months, which is substantially longer than the approximately 12 months previously reported for patients with advanced NSCLC and cancer cachexia treated with chemotherapy [8, 9, 19]. Although direct comparison with previous retrospective cohorts is not methodologically appropriate given differences in study design and patient populations, the median OS observed in our study appears favourable in the context of previously reported outcomes in this population. In particular, a recent study in a similar population of patients with advanced NSCLC receiving chemoimmunotherapy, in which cachexia was defined based on body weight loss alone, reported a median OS of 10.8 months, which is numerically lower than that observed in our cohort [9]. Several factors likely contributed to this favourable outcome. Cachexia was ameliorated in a large proportion of patients, with 71.8% of them transitioning to a non‐cachectic state at 12 weeks and 82.0% at 24 weeks. Improvements in patient‐reported outcomes provide additional support for the adoption of this treatment, with significant gains in the QOL‐ACD appetite and face scale scores. This is in agreement with the effects of anamorelin on appetite and patients' subjective well‐being [10, 11]. In contrast, social attitude scores declined, highlighting the importance of integrated psychosocial support alongside pharmacological interventions. Because anamorelin may improve appetite and meal enjoyment, nutritional counselling may help patients benefit from these effects and further improve quality of life. Anamorelin did not lead to significant weight gain in our study. However, a substantial proportion of patients transitioned to a non‐cachectic state. Cancer cachexia was defined using a multidimensional framework that includes not only body weight but also clinical symptoms and laboratory parameters. Improvements in these components may occur independently of measurable weight gain. Previous studies have shown that patients with advanced NSCLC associated with cancer cachexia frequently experience substantial weight loss during chemotherapy, with or without ICIs, particularly within the first 6 weeks of treatment [20]. Notably, weight loss, recognized as a poor prognostic factor in NSCLC, was not significant in the participants of this study [21, 22]. Prevention of weight loss may have helped preserve treatment tolerance and enabled subsequent therapy, thereby contributing to prolonged survival. Indeed, 54.2% of patients in our study received subsequent treatment, a relatively high rate compared with the general NSCLC subpopulation, and especially notable given the initial cachectic status of the cohort. Given that body weight changes during chemotherapy are heterogeneous, with weight gain observed in a subset of patients with NSCLC, the interpretation of body weight as a surrogate marker remains complex [23, 24]. In our cohort, although no significant weight gain was observed, the absence of further weight loss and the high rate of cachexia reversal may still reflect a clinically meaningful benefit.

Landmark analyses further demonstrated that patients who transitioned to a non‐cachectic state achieved significantly longer OS compared with those who remained cachectic, whereas no differences were observed in PFS. These results suggest that it was the reversal of cachexia rather than the tumour response itself that determined the prognosis. However, no baseline predictors of cachexia reversal were identified, highlighting the need for further research to define patient subgroups most likely to benefit from anamorelin treatment. It is worth noting that prolonged administration of anamorelin (≥ 12 weeks) did not translate into improved survival, indicating that the key determinant was not treatment duration but rather whether cachexia was ameliorated or not. This finding highlights that the clinical value of anamorelin treatment lies in achieving cachexia reversal, not in the length of exposure. The duration of anamorelin treatment in our study (median: 13.15 weeks) was longer than that reported in real‐world post‐marketing surveillance in Japan (median: 29 days) [25]. This difference may be the result of a study design in which physicians were encouraged to continue treatment unless clear intolerance or lack of benefit was observed, as well as the selective recruitment of patients with relatively good performance status who were scheduled to receive chemoimmunotherapy.

Although AEs were frequently observed in this study, no new safety signals were identified with the addition of anamorelin to chemoimmunotherapy. However, grade ≥ 3 hyperglycemia occurred in 5.1% of patients, with half of them having pre‐existing diabetes. Given that dexamethasone is routinely co‐administered as an antiemetic during induction of chemoimmunotherapy, its combination with anamorelin may exacerbate hyperglycemia, warranting careful glucose monitoring in at‐risk patients. Importantly, fatigue (83.9%) and malaise (87.3%) were frequently observed; however, these symptoms are included in the diagnostic criteria for cancer cachexia and were likely present at baseline. Nevertheless, a potential contribution of treatment‐related toxicity cannot be excluded. Dyspnea may similarly reflect the underlying disease. Therefore, interpretation of symptom‐based AEs is limited in patients with cancer cachexia. Overall, the safety profile observed in this study was considered acceptable, supporting the feasibility of combining anamorelin with chemoimmunotherapy in this patient population.

This study has several strengths, including its prospective design, focus on a subpopulation with advanced NSCLC associated with cancer cachexia (which has been frequently excluded from clinical trial participation) and a relatively large sample size.

Limitations include the single‐arm, non‐randomized design; the potential inter‐institutional variability in cachexia assessment; and the fact that the study cohort was composed exclusively of Japanese individuals, which may limit the generalizability of the results.

Post hoc analyses of the ONO‐7643‐04 study suggested that anamorelin may be more beneficial when administered earlier in the treatment course, and that improvements in appetite are more likely in patients receiving cytotoxic chemotherapy [26]. The results of that trial support the use of anamorelin in combination with chemoimmunotherapy for patients with advanced NSCLC and cancer cachexia.

In conclusion, the addition of anamorelin to chemoimmunotherapy did not improve PFS in patients with advanced NSCLC associated with cancer cachexia. However, cachexia reversal was observed in a high proportion of patients, and this was strongly associated with prolonged OS. These findings suggest that the clinical value of anamorelin lies in its ability to achieve cachexia reversal, which may ultimately translate into a survival benefit. Future trials should consider OS, rather than PFS, as the primary endpoint, and validation studies are needed to establish cachexia reversal as a surrogate marker for survival. Cancer cachexia represents a severe condition encountered not only in patients with lung cancer but also across a wide range of malignancies. Accordingly, the findings of this study may hold relevance beyond lung cancer. In addition to anamorelin, novel therapeutic strategies against cachexia, including an antibody against growth differentiation factor 15, are currently under development [27]. The emergence of therapeutic strategies against cachexia, which has long been regarded as the ‘last illness’, is highly encouraging.

## Funding

This study was sponsored by Ono Pharmaceutical Co. Ltd.

## Ethics Statement

Ethical approval for this study was granted by the Institutional Review Board of the Nonprofit Organization Clinical Trial Network Fukuoka on 4 August 2021 (number: 21‐E02).

## Consent

In accordance with the Declaration of Helsinki, written informed consent for participation and publication was obtained from all the patients prior to enrolment.

## Conflicts of Interest

Makoto Hibino received speaking honoraria from AstraZeneca, Boehringer Ingelheim, Bristol‐Myers, Chugai Pharmaceutical Co. Ltd. and Eli Lilly. Hiroyasu Kaneda received speaking honoraria from AstraZeneca, Bristol‐Myers, Chugai Pharmaceutical Co. Ltd., MSD and Ono Pharmaceutical Co. Ltd. Tadaaki Yamada received grants from Eli Lilly Japan K.K. and Taiyo Kagaku Co. Ltd., and personal fees from Eli Lilly Japan K.K. and Chugai Pharmaceutical Co. Ltd., outside the submitted work. Koichi Takayama received grants from Chugai‐Roche, Taiho Pharmaceutical, Boehringer Ingelheim and Ono Pharmaceutical, consulting fees from Ono Pharmaceutical and AstraZeneca, and lecture fees from AstraZeneca, Ono Pharmaceutical, Chugai‐Roche, Eli Lilly and Boehringer Ingelheim. The other authors declare no conflicts of interest.

## Supporting information


**Table S1:** Detail of the treatment regimens (*N* = 114).
**Table S2:** Data information.
**Table S3:** Changes in ECOG‐PS from baseline.
**Table S4:** Treatment response.
**Table S5:** Treatment response stratified by PD‐L1 TPS.
**Table S6:** Logistic regression analysis for baseline factors predictive of cancer cachexia at week.12.
**Table S7:** Logistic regression analysis for baseline factors predictive of cancer cachexia at week 24.
**Figure S1:** Changes in QOL‐ACD scores from baseline.
**Figure S2:** Changes in body weight and laboratory values from baseline.
**Figure S3:** Reasons for discontinuation of anamorelin. (A) Overall population. (B) Reasons for discontinuation within 0–12 weeks. (C) Reasons for discontinuation after 12 weeks.
**Figure S4:** Kaplan–Meier survival curves stratified by PD‐L1 expression level (≥ 50% vs. < 50%) for (A) TTF, (B) PFS, and (C) OS. CI, confidence interval; NE, not estimable; OS, overall survival; PD‐L1, programmed death‐ligand 1; PFS, progression‐free survival; TTF, time to treatment failure.
**Figure S5:** Landmark analysis of Kaplan–Meier curves for (A) PFS and (B) OS in patients treated with anamorelin for ≥ 12 weeks versus < 12 weeks. OS; overall survival; PFS, progression‐free survival.
**Figure S6:** Reasons for chemoimmunotherapy discontinuation.

## Data Availability

The datasets generated during the current study are not publicly available because of ethical constraints, but are available from the corresponding author upon reasonable request.
