# Correction to ‘A Prospective Observational Study of Anamorelin During Chemoimmunotherapy in Non‐Small Cell Lung Cancer With Cachexia (SPIRAL‐ANA)’

**DOI:** 10.1002/jcsm.70352

**Published:** 2026-08-04

Morimoto K , Uchino J , Hibino M , et al., “A Prospective Observational Study of Anamorelin During Chemoimmunotherapy in Non‐Small Cell Lung Cancer With Cachexia (SPIRAL‐ANA),” Journal of Cachexia, Sarcopenia and Muscle 17, no. 4 (2026): e70340, https://onlinelibrary.wiley.com/doi/10.1002/jcsm.70340.42411631 10.1002/jcsm.70340PMC13339061

In the published version, the second sentence of the Acknowledgements section reads:

Data management and monitoring for this study were conducted by CReS Kyushu under a funding contract with Chugai Pharmaceutical Co. Ltd.

The correct sentence should be:

Data management and monitoring for this study were conducted by CReS Kyushu under a funding contract with Ono Pharmaceutical Co. Ltd.

We apologize for this mistake.

