# Supplementary material for: A Prospective Observational Study of Anamorelin During Chemoimmunotherapy in Non‐Small Cell Lung Cancer With Cachexia (SPIRAL‐ANA)
Source: J Cachexia Sarcopenia Muscle. 2026 Jul 7;17(4):e70340. doi: 10.1002/jcsm.70340 (PMC13339061; doi:10.1002/jcsm.70340)

**Supplementary Material**

**Table of contents**

Supplementary Table 1………………………………………………………………………………………………………….. page 2

Supplementary Table 2 …………………………………………………………………………………………………………. page 3

Supplementary Table 3 …………………………………………………………………………………………………………. page 5

Supplementary Table 4 …………………………………………………………………………………………………………. page 6

Supplementary Table 5 …………………………………………………………………………………………………………. page 7

Supplementary Table 6 …………………………………………………………………………………………………………. page 8

Supplementary Table 7 …………………………………………………………………………………………………………. page 10

Supplementary Figure 1 …………………………………………………………………………………………………………page 12

Supplementary Figure 2 …………………………………………………………………………………………………………page 13

Supplementary Figure 3 …………………………………………………………………………………………………………page 14

Supplementary Figure 4 …………………………………………………………………………………………………………page 15

Supplementary Figure 5 …………………………………………………………………………………………………………page 16

Supplementary Figure 6 …………………………………………………………………………………………………………page 17

**Supplementary Table 1. Detail of the treatment regimens (N = 114)**

| **Treatment regimen** | **N** | **%** |
| --- | --- | --- |
| Carboplatin + pembrolizumab + nab-paclitaxel | 35 | 30.7 |
| Carboplatin + pemetrexed + pembrolizumab | 31 | 27.2 |
| Carboplatin+ nab-paclitaxel + atezolizumab | 15 | 13.2 |
| Carboplatin + paclitaxel + bevacizumab + atezolizumab | 7 | 6.1 |
| Carboplatin + paclitaxel + pembrolizumab | 6 | 5.3 |
| Nivolumab + ipilimumab | 4 | 3.5 |
| Carboplatin + paclitaxel + nivolumab + ipilimumab | 3 | 2.6 |
| Cisplatin + pemetrexed + pembrolizumab | 3 | 2.6 |
| Carboplatin + pemetrexed + nivolumab + ipilimumab | 2 | 1.8 |
| Carboplatin + nab-paclitaxel + durvalumab + tremelimumab | 2 | 1.8 |
| Carboplatin + pemetrexed + atezolizumab | 1 | 0.9 |
| Carboplatin + pemetrexed + durvalumab + tremelimumab | 1 | 0.9 |
| Carboplatin + pemetrexed + nab-paclitaxel + atezolizumab | 1 | 0.9 |
| Cisplatin + pemetrexed + nivolumab + ipilimumab | 1 | 0.9 |
| Nab-paclitaxel + atezolizumab | 1 | 0.9 |
| Pemetrexed + pembrolizumab | 1 | 0.9 |

**Supplementary Table 2**.

**Inclusion criteria**

1. aged 20 years or older at the time of informed consent.
2. scheduled to receive chemoimmunotherapy, with initiation of anamorelin administration planned between 7 days prior to and the day of the first chemoimmunotherapy dose.
3. histologically or cytologically confirmed NSCLC.
4. unresectable stage III/IV disease or postoperative recurrence not amenable to curative resection or radiotherapy.
5. patients with driver gene alterations, excluding those with KRAS G12C mutations and EGFR exon 20 insertions, must have received prior treatment with kinase inhibitors. In patients with non-squamous NSCLC, testing for EGFR mutations is mandatory, while testing for ALK rearrangements, ROS1 rearrangements, and BRAF mutations is recommended whenever feasible.
6. no prior systemic cytotoxic chemotherapy for advanced or recurrent disease (prior adjuvant or neoadjuvant chemotherapy is permitted).
7. meeting the indication criteria for anamorelin: patients with ≥5% body weight loss within 6 months and anorexia, along with ≥2 of the following:
8. fatigue or malaise (≥ grade 1 according to CTCAE).
9. generalized muscle weakness (≥ grade 1, assessed by grip strength, walking speed, chair stand test, or other indicators).
10. laboratory abnormalities: CRP > 0.5 mg/dL, albumin < 3.2 g/dL, or hemoglobin < 12 g/dL.
11. at least one measurable lesion according to RECIST v1.1 criteria.
12. ECOG performance status of 0–2.
13. expected survival for at least 3 months.
14. willing to provide written informed consent.

**Exclusion criteria**

1. contraindications to anamorelin administration, including:
2. congestive heart failure.
3. history of myocardial infarction or angina pectoris.
4. severe conduction abnormalities (e.g., complete atrioventricular block).
5. concomitant treatment with clarithromycin, indinavir, itraconazole, nelfinavir, saquinavir, telaprevir, voriconazole, ritonavir-containing agents, or cobicistat-containing agents.
6. moderate to severe hepatic impairment (Child-Pugh class B or C).
7. known hypersensitivity to components of anamorelin.
8. gastrointestinal obstruction or other structural abnormalities precluding oral intake.
9. uncontrolled diabetes mellitus.
10. refractory nausea and vomiting, chronic gastrointestinal disorders, inability to swallow the study drug, or prior gastrointestinal surgery likely to impair absorption of anamorelin.
11. any other condition judged by the investigator to render the patient inappropriate for participation in this study.

**Supplementary Table 3. Changes in ECOG-PS from baseline.**

|  | Baseline | | Week 3 | | Week 12 | | Week 24 | |
| --- | --- | --- | --- | --- | --- | --- | --- | --- |
| ECOG-PS | N | % | N | % | N | % | N | % |
| 0 | 13 | 11.4 | 18 | 16.4 | 14 | 14.7 | 7 | 9.7 |
| 1 | 81 | 71.1 | 73 | 66.4 | 63 | 66.3 | 55 | 76.4 |
| 2 | 20 | 17.5 | 13 | 11.8 | 12 | 12.6 | 10 | 13.9 |
| 3 | . | . | 4 | 3.6 | 4 | 4.2 | . | . |
| 4 | . | . | 2 | 1.8 | 2 | 2.1 | . | . |

ECOG-PS, Eastern Cooperative Oncology Group performance status.

**Supplementary Table 4. Treatment response**

| Response to treatment | Number of patients | % |
| --- | --- | --- |
| Complete response | 1 | 0.9 |
| Partial response | 65 | 57.0 |
| Stable disease | 26 | 22.8 |
| Progressive disease | 18 | 15.8 |
| Not evaluable | 4 | 3.5 |
| Complete response rate (95%CI) | 0.9% (0.2–4.8%) |  |
| Objective response rate (95% CI) | 57.9% (48.7–66.6%) |  |

CI, confidence interval

**Supplementary Table 5. Treatment response stratified by PD-L1 TPS**

| **Response to treatment** | **PD-L1 TPS <50%** | | **PD-L1 TPS ≥50%** | | **P value** |
| --- | --- | --- | --- | --- | --- |
|  | **n (%)** | **95%CI** | **n (%)** | **95%CI** |  |
| CR | 1 (1.4) |  | 0 (0.0) |  |  |
| PR | 36 (49.3) |  | 28 (75.7) |  |  |
| SD | 22 (30.1) |  | 3 (8.1) |  |  |
| PD | 11 (15.1) |  | 5 (13.5) |  |  |
| NE | 3 (4.1) |  | 1 (2.7) |  |  |
|  |  |  |  |  |  |
| CRR | 1 (1.4) | 0.2-7.4 | 0 (0.0) | 0.0-9.4 | 0.474 |
| ORR | 37 (50.7) | 39.5-61.8 | 28 ( 75.7) | 59.9-86.6 | 0.012 |

CI, confidence interval; CR, complete response; CRR, complete response rate; NE, not evaluable; ORR; objective response rate; PD, progressive disease; PD-L1 TPS, programmed death-ligand 1 tumor proportion score; PR, partial response; SD, stable disease.

**Supplementary Table 6. Logistic regression analysis for baseline factors predictive of cancer cachexia at week 12**

|  | | Total | | Cancer cachexia | | Odds ratio | |  |
| --- | --- | --- | --- | --- | --- | --- | --- | --- |
| Subgroup | Category | N | % | N | % | Point estimates (95%CI) | P value | |
| Age (years) | <75 | 52 | 61.2 | 18 | 34.6 | 1 (Reference) | 0.106 | |
|  | ≥75 | 33 | 38.8 | 6 | 18.2 | 0.420 (0.146-1.203) |  | |
| Sex | Male | 64 | 75.3 | 17 | 26.6 | 1 (Reference) | 0.551 | |
|  | Female | 21 | 24.7 | 7 | 33.3 | 1.382 (0.477-4.004) |  | |
| ECOG-PS | 0/1 | 70 | 82.4 | 20 | 28.6 | 1 (Reference) | 0.882 | |
|  | 2 | 15 | 17.6 | 4 | 26.7 | 0.909 (0.259-3.193) |  | |
| Stage | III | 6 | 7.1 | 1 | 16.7 | 1 (Reference) | 0.426 | |
|  | IV | 73 | 85.9 | 20 | 27.4 | 1.887 (0.207-17.161) |  | |
|  | Postoperative recurrence | 6 | 7.1 | 3 | 50.0 | 5.000 (0.344-72.766) |  | |
| BMI (kg/m^2^) | <20 | 37 | 43.5 | 12 | 32.4 | 1 (Reference) | 0.4514 | |
|  | ≥20 | 48 | 56.5 | 12 | 25.0 | 0.694 (0.269-1.794) |  | |
| PD-L1 TPS (%) | <50 | 51 | 60.0 | 14 | 27.5 | 1 (Reference) | 0.643 | |
|  | ≥50 | 31 | 36.5 | 10 | 32.3 | 1.259 (0.476-3.327) |  | |
| Histology | Adenocarcinoma | 38 | 44.7 | 9 | 23.7 | 1 (Reference) | 0.403 | |
|  | Others | 47 | 55.3 | 15 | 31.9 | 1.510 (0.574-3.973) |  | |
| Smoking status | Never smoker | 12 | 14.1 | 3 | 25.0 | 1 (Reference) | 0.716 | |
|  | Current smoker | 23 | 27.1 | 8 | 34.8 | 1.600 (0.335-7.639) |  | |
|  | Former smoker | 50 | 58.8 | 13 | 26.0 | 1.054 (0.247-4.500) |  | |
| Fatigue or malaise | Yes | 83 | 97.6 | 24 | 28.9 | 1 (Reference) | 0.983 | |
|  | No | 2 | 2.4 | 0 | 0 | <0.001 (not estimable) |  | |
| Generalized muscle weakness | Yes | 54 | 63.5 | 18 | 33.3 | 1 (Reference) | 0.173 | |
|  | No | 31 | 36.5 | 6 | 19.4 | 0.480 (0.167-1.379) |  | |
| Hb (g/dL) | ≥12 | 42 | 49.4 | 9 | 21.4 | 1 (Reference) | 0.172 | |
|  | <12 | 43 | 50.6 | 15 | 34.9 | 1.964 (0.746-5.170) |  | |
| Alb (g/dL) | ≥3.2 | 53 | 62.4 | 13 | 24.5 | 1 (Reference) | 0.330 | |
|  | <3.2 | 32 | 37.6 | 11 | 34.4 | 1.612 (0.616-4.214) |  | |
| CRP (mg/dL) | ≤0.5 | 18 | 21.2 | 4 | 22.2 | 1 (Reference) | 0.525 | |
|  | >0.5 | 67 | 78.8 | 20 | 29.9 | 1.489 (0.436-5.086) |  | |
| Baseline total tumor size (mm) | Per 10 mm increase | - | - | - | - | 1.045 (0.947-1.153) | 0.3814 | |

Alb, albumin; BMI, body mass index; CRP, C-reactive protein; ECOG-PS, Eastern Cooperative Oncology Group performance status; Hb, hemoglobin; PD-L1 TPS, programmed death-ligand 1 tumor proportion score.

**Supplementary Table 7. Logistic regression analysis for baseline factors predictive of cancer cachexia at week 24**

|  | | Total | | Cancer cachexia | | Odds ratio | |
| --- | --- | --- | --- | --- | --- | --- | --- |
| Subgroup | Category | N | % | N | % | Point estimates (95%CI) | P value |
| Age (years) | <75 | 40 | 65.6 | 10 | 25.0 | 1 (Reference) | 0.081 |
|  | ≥75 | 21 | 34.4 | 1 | 4.8 | 0.150 (0.018-1.265) |  |
| Sex | Male | 46 | 75.4 | 9 | 19.6 | 1 (Reference) | 0.588 |
|  | Female | 15 | 24.6 | 2 | 13.3 | 0.632 (0.121-3.318) |  |
| ECOG-PS | 0/1 | 52 | 85.2 | 10 | 19.2 | 1 (Reference) | 0.564 |
|  | 2 | 9 | 14.8 | 1 | 11.1 | 0.525 (0.059-4.692) |  |
| Stage | III | 4 | 6.6 | 0 | 0.0 | 1 (Reference) | 0.329 |
|  | IV | 53 | 86.9 | 9 | 17.0 | >999.999 |  |
|  | Postoperative recurrence | 4 | 6.6 | 2 | 50.0 | >999.999 |  |
| BMI (kg/m^2^) | <20 | 26 | 42.6 | 4 | 15.4 | 1 (Reference) | 0.6437 |
|  | ≥20 | 35 | 57.4 | 7 | 20.0 | 1.375 (0.357-5.301) |  |
| PD-L1 TPS (%) | <50 | 39 | 63.9 | 8 | 20.5 | 1 (Reference) | 0.554 |
|  | ≥50 | 21 | 34.4 | 3 | 14.3 | 0.646 (0.152-2.749) |  |
| Histology | Adenocarcinoma | 30 | 49.2 | 6 | 20.0 | 1 (Reference) | 0.695 |
|  | Others | 31 | 50.8 | 5 | 16.1 | 0.769 (0.208-2.851) |  |
| Smoking status | Never smoker | 9 | 14.8 | 2 | 22.2 | 1 (Reference) | 0.788 |
|  | Current smoker | 16 | 26.2 | 2 | 12.5 | 0.500 (0.058-4.335) |  |
|  | Former smoker | 36 | 59.0 | 7 | 19.4 | 0.845 (0.143-4.985) |  |
| Fatigue or malaise | Yes | 60 | 98.4 | 11 | 18.3 | 1 (Reference) | 0.985 |
|  | No | 1 | 1.6 | 0 | 0 | <0.001 (not estimable) |  |
| Generalized muscle weakness | Yes | 32 | 52.5 | 8 | 25.0 | 1 (Reference) | 0.148 |
|  | No | 29 | 47.5 | 3 | 10.3 | 0.346 (0.082-1.458) |  |
| Hb (g/dL) | ≥12 | 30 | 49.2 | 3 | 10.0 | 1 (Reference) | 0.120 |
|  | <12 | 31 | 50.8 | 8 | 25.8 | 3.130 (0.743-13.196) |  |
| Alb (g/dL) | ≥3.2 | 39 | 63.9 | 8 | 20.5 | 1 (Reference) | 0.505 |
|  | <3.2 | 22 | 36.1 | 3 | 13.6 | 0.612 (0.144-2.594) |  |
| CRP (mg/dL) | ≤0.5 | 15 | 24.6 | 2 | 13.3 | 1 (Reference) | 0.588 |
|  | >0.5 | 46 | 75.4 | 9 | 19.6 | 1.581 (0.301-8.294) |  |
| Baseline total tumor size (mm) | Per 10 mm increase | - | - | - | - | 1.041 (0.907-1.195) | 0.5691 |

Alb, albumin; BMI, body mass index; CRP, C-reactive protein; ECOG-PS, Eastern Cooperative Oncology Group performance status; Hb, hemoglobin; PD-L1 TPS, programmed death-ligand 1 tumor proportion score.

**Supplementary Figure 1.** Changes in QOL-ACD scores from baseline.

**Supplementary Figure 2.** Changes in body weight and laboratory values from baseline.

**Supplementary Figure 3.** Reasons for discontinuation of anamorelin. (A) Overall population. (B) Reasons for discontinuation within 0–12 weeks. (C) Reasons for discontinuation after 12 weeks.


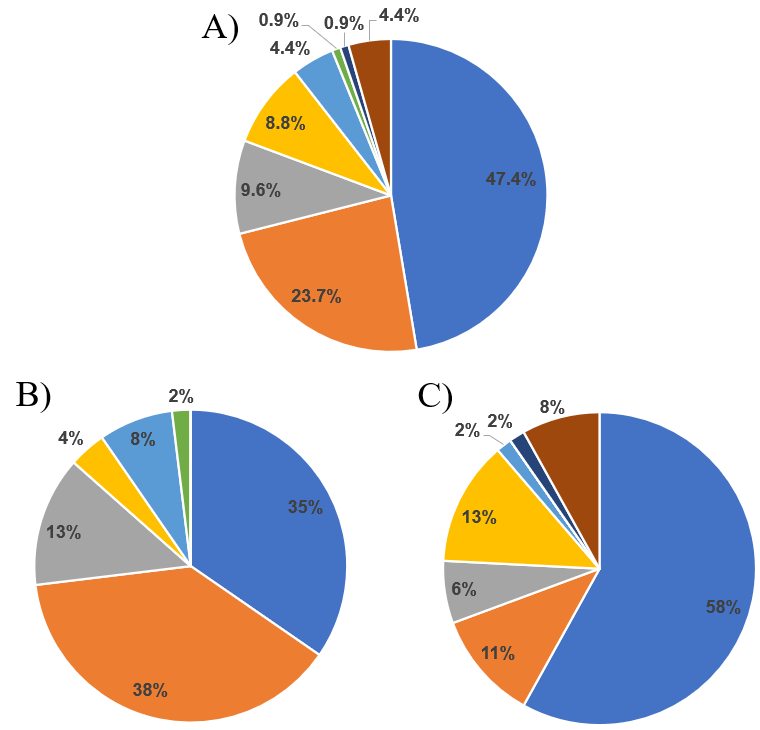


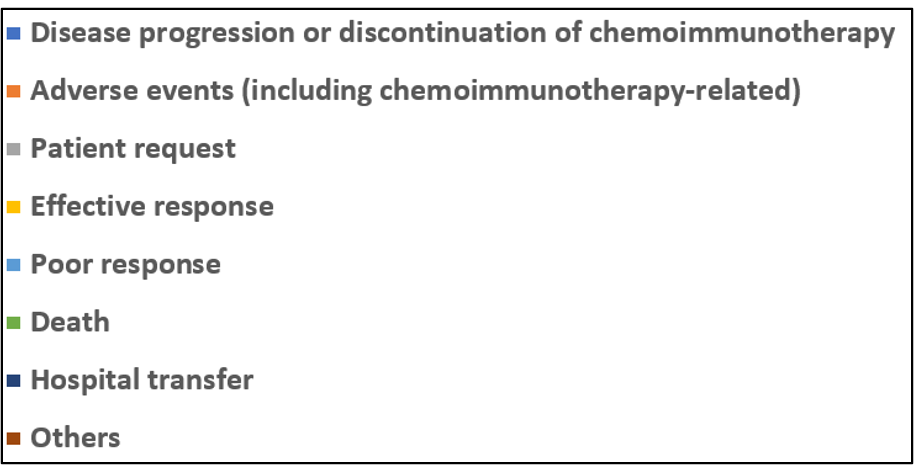


**Supplementary Figure 4.** Kaplan–Meier survival curves stratified by PD-L1 expression level (≥50% vs. <50%) for (A) TTF, (B) PFS, and (C) OS. CI, confidence interval; NE, not estimable; OS, overall survival; PD-L1, programmed death-ligand 1; PFS, progression-free survival; TTF, time to treatment failure.


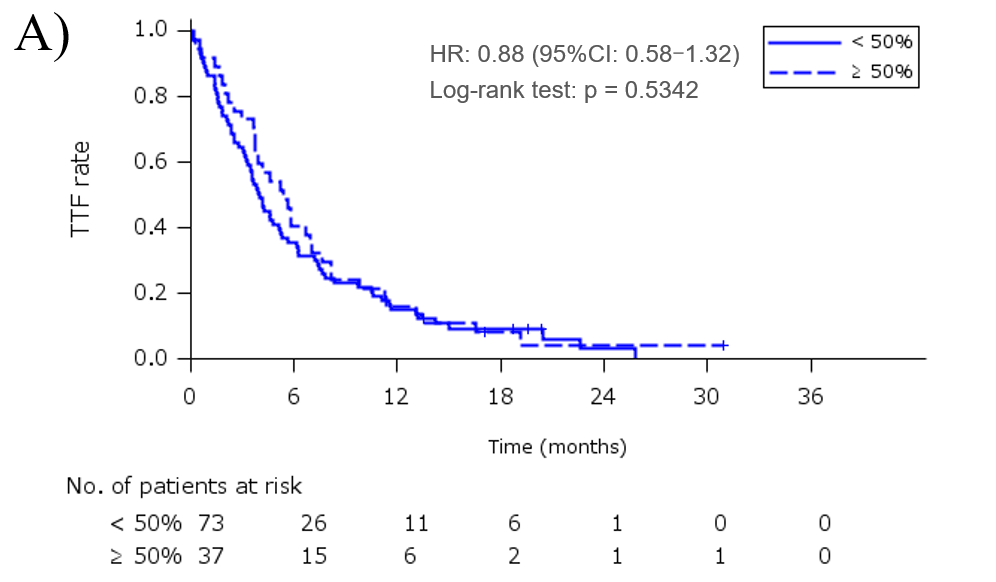


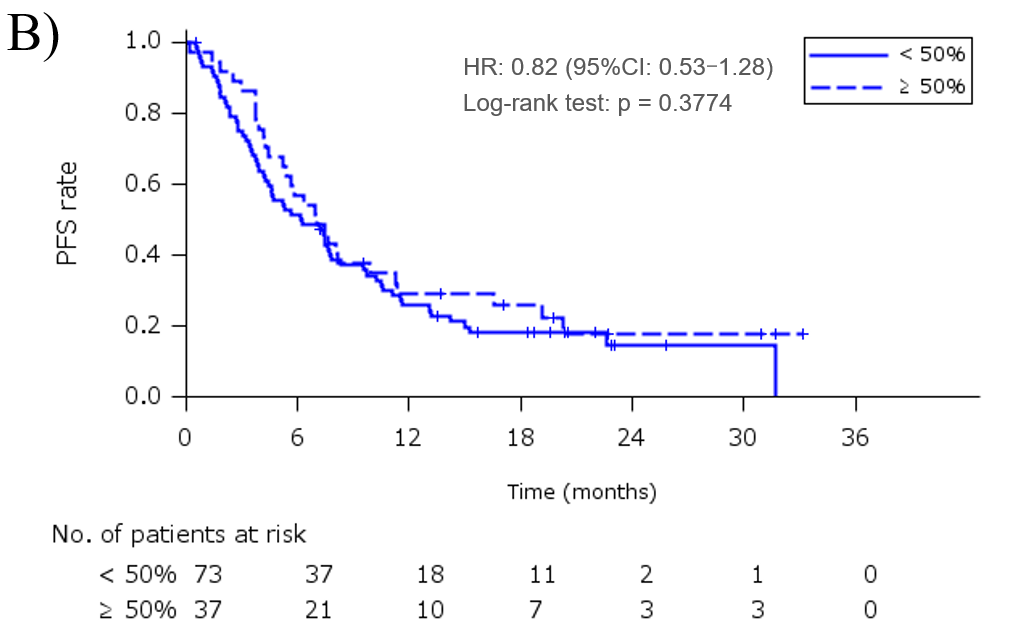


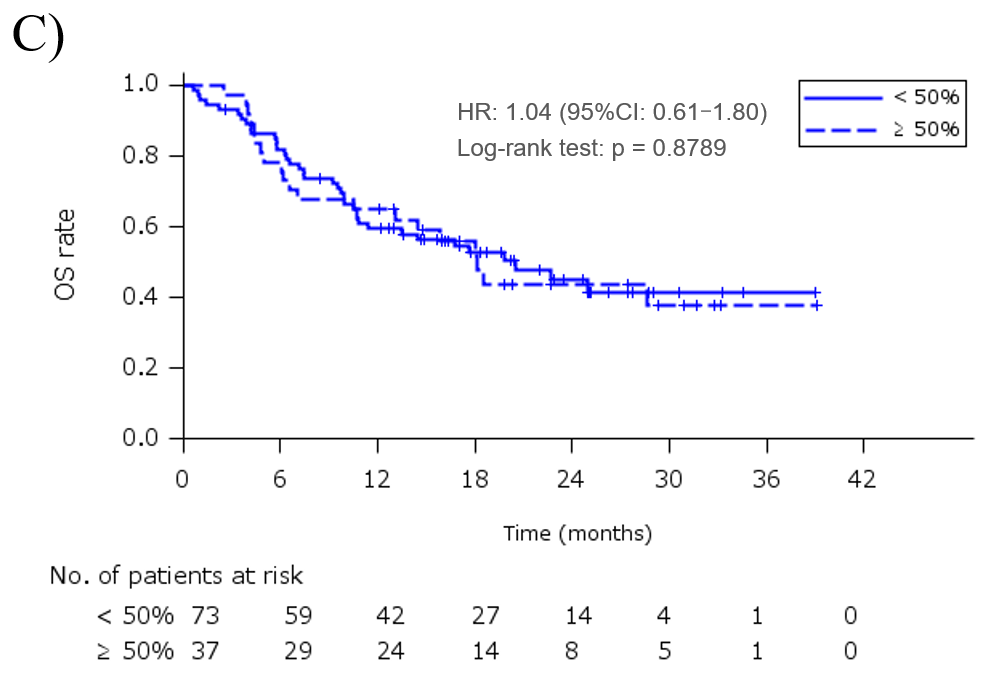


**Supplementary Figure 5**. Landmark analysis of Kaplan–Meier curves for (A) PFS and (B) OS in patients treated with anamorelin for ≥12 weeks versus <12 weeks. OS; overall survival; PFS, progression-free survival.


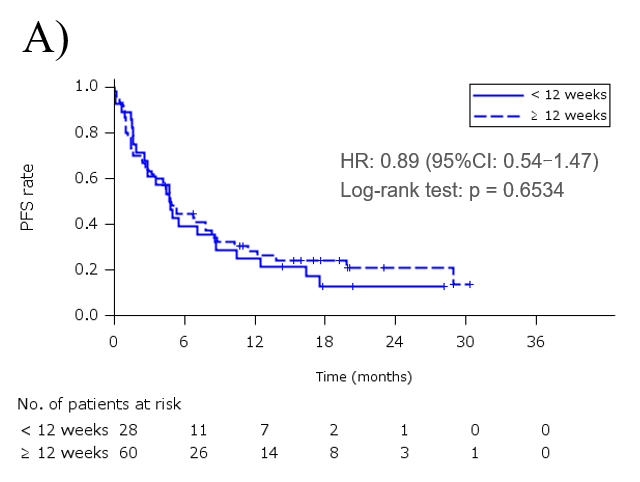


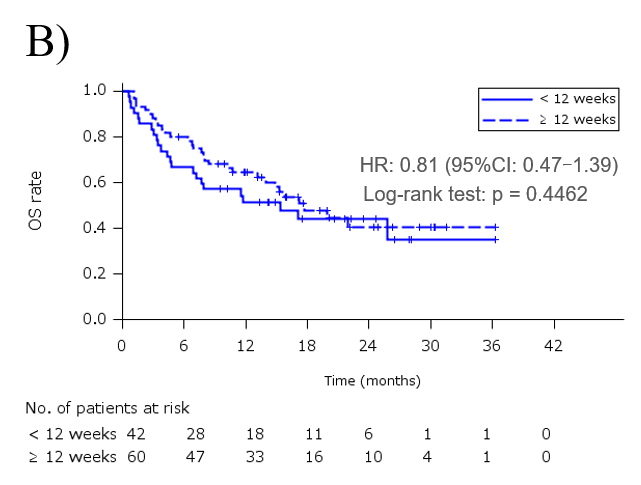


**Supplementary Figure 6**. Reasons for chemoimmunotherapy discontinuation


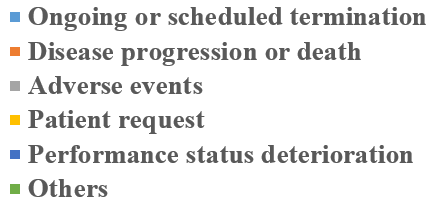

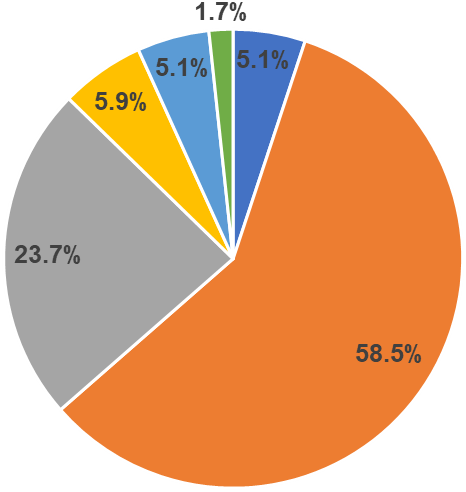

Supplement: Supplementary file 1 — Table S1: Detail of the treatment regimens (N = 114). Table S2: Data information. Table S3: Changes in ECOG‐PS from baseline. Table S4: Treatment response. Table S5: Treatment response stratified by PD‐L1 TPS. Table S6: Logistic regression analysis for baseline factors predictive of cancer cachexia at week.12. Table S7: Logistic regression analysis for baseline factors predictive of cancer cachexia at week 24. Figure S1: Changes in QOL‐ACD scores from baseline. Figure S2: Changes in body weight and laboratory values from baseline. Figure S3: Reasons for discontinuation of anamorelin. (A) Overall population. (B) Reasons for discontinuation within 0–12 weeks. (C) Reasons for discontinuation after 12 weeks. Figure S4: Kaplan–Meier survival curves stratified by PD‐L1 expression level (≥ 50% vs. < 50%) for (A) TTF, (B) PFS, and (C) OS. CI, confidence interval; NE, not estimable; OS, overall survival; PD‐L1, programmed death‐ligand 1; PFS, progression‐free survival; TTF, time to treatment failure. Figure S5: Landmark analysis of Kaplan–Meier curves for (A) PFS and (B) OS in patients treated with anamorelin for ≥ 12 weeks versus < 12 weeks. OS; overall survival; PFS, progression‐free survival. Figure S6: Reasons for chemoimmunotherapy discontinuation. [file JCSM-17-e70340-s001.docx]
